# Roles of RAGE/ROCK1 Pathway in HMGB1-Induced Early Changes in Barrier Permeability of Human Pulmonary Microvascular Endothelial Cell

**DOI:** 10.3389/fimmu.2021.697071

**Published:** 2021-10-20

**Authors:** Meng-jiao Zhao, Hao-ran Jiang, Jing-wen Sun, Zi-ang Wang, Bo Hu, Cheng-rui Zhu, Xiao-han Yin, Ming-ming Chen, Xiao-chun Ma, Wei-dong Zhao, Zheng-gang Luan

**Affiliations:** ^1^ Department of Critical Care Medicine, The First Affiliated Hospital of China Medical University, Shenyang, China; ^2^ Department of Breast Oncology, The First Affiliated Hospital of China Medical University, Shenyang, China; ^3^ Department of Developmental Cell Biology, Key Laboratory of Cell Biology, Ministry of Public Health, and Key Laboratory of Medical Cell Biology, Ministry of Education, China Medical University, Shenyang, China

**Keywords:** endothelium, high mobility group box 1, barrier permeability, signaling, inflammation

## Abstract

**Background:**

High mobility group box 1 (HMGB1) causes microvascular endothelial cell barrier dysfunction during acute lung injury (ALI) in sepsis, but the mechanisms have not been well understood. We studied the roles of RAGE and Rho kinase 1 (ROCK1) in HMGB1-induced human pulmonary endothelial barrier disruption.

**Methods:**

In the present study, the recombinant human high mobility group box 1 (rhHMGB1) was used to stimulate human pulmonary microvascular endothelial cells (HPMECs). The endothelial cell (EC) barrier permeability was examined by detecting FITC-dextran flux. CCK-8 assay was used to detect cell viability under rhHMGB1 treatments. The expression of related molecules involved in RhoA/ROCK1 pathway, phosphorylation of myosin light chain (MLC), F-actin, VE-cadherin and ZO-1 of different treated groups were measured by pull-down assay, western blot and immunofluorescence. Furthermore, we studied the effects of Rho kinase inhibitor (Y-27632), ROCK1/2 siRNA, RAGE-specific blocker (FPS-ZM1) and RAGE siRNA on endothelial barrier properties to elucidate the related mechanisms.

**Results:**

In the present study, we demonstrated that rhHMGB1 induced EC barrier hyperpermeability in a dose-dependent and time-dependent manner by measuring FITC-dextran flux, a reflection of the loss of EC barrier integrity. Moreover, rhHMGB1 induced a dose-dependent and time-dependent increases in paracellular gap formation accompanied by the development of stress fiber rearrangement and disruption of VE-cadherin and ZO-1, a phenotypic change related to increased endothelial contractility and endothelial barrier permeability. Using inhibitors and siRNAs directed against RAGE and ROCK1/2, we systematically determined that RAGE mediated the rhHMGB1-induced stress fiber reorganization *via* RhoA/ROCK1 signaling activation and the subsequent MLC phosphorylation in ECs.

**Conclusion:**

HMGB1 is capable of disrupting the endothelial barrier integrity. This study demonstrates that HMGB1 activates RhoA/ROCK1 pathway *via* RAGE, which phosphorylates MLC inducing stress fiber formation at short time, and HMGB1/RAGE reduces AJ/TJ expression at long term independently of RhoA/ROCK1 signaling pathway.

## Introduction

A hallmark of acute lung injury (ALI) is pulmonary edema caused by increased vascular permeability during septic inflammation (1, 2). The pulmonary microvascular endothelial cells play a key effect on maintaining the endothelial barrier integrity between the microvascular lumen and the lung interstitium. EC barrier function depends on the integrity of endothelial cell (EC), the coordinate expression and interplay of proteins in cellular junction complexes, including the F-actin cytoskeleton, adherens junction (AJ) and tight junction (TJ) (3, 4). The barrier hyperpermeability is always related to the cytoskeleton rearrangement of EC and the disruption of AJ and TJ, resulting in EC contraction and intercellular gap formation (5, 6). High mobility group box 1 (HMGB1), a potent proinflammatory cytokine, can disrupt intercellular junctions, increasing endothelial barrier permeability (7, 8). Our previous investigation shows that HMGB1 is involved in the progression of ALI, which has been demonstrated to be associated with microvascular barrier dysfunction elicited by the AJ and TJ disruption (7, 9). Recent studies have indicated that HMGB1 and the receptor for advanced glycation end products (RAGE) conduce to endothelial barrier dysfunction, and RAGE is the primary receptor mediating HMGB1-induced hyperpermeability and paracellular gap formation (10–12). HMGB1 also elicits activation of Rho small GTPases which play important effects on rearranging the F-actin cytoskeleton and the intercellular junctional proteins (13, 14). Rho kinase (ROCK) is a downstream target of RhoA and exists in two similar isoforms: ROCK1 and ROCK2 (15, 16). ROCK activated by the GTP-bound form of Rho can directly phosphorylate myosin light chain (MLC) and reduce the dephosphorylation of phosphorylated MLC (pMLC) (17, 18). ROCK1 and ROCK2 play different functional roles in regulating cytoskeleton arrangement by phosphorylating different downstream proteins (16). The previous studies have demonstrated that ROCK1 was involved in TNFα-induced early endothelial hyperpermeability and ROCK1 induced actin cytoskeletal instability by regulating actomyosin contraction, whereas ROCK2 stabilized the actin cytoskeleton by regulating cofilin phosphorylation (15, 16). To verify the hypothesis that ROCK-dependent cell contraction plays a key role in HMGB1-induced increases in pulmonary microvascular endothelial barrier permeability, we investigated the effects of HMGB1 on the structure and function of endothelial barrier and elucidated the roles of RAGE and ROCK in HMGB1-induced human pulmonary endothelial barrier disruption. Our findings indicated that HMGB1 induced F-actin rearrangement, AJ/TJ rupture, and then enhanced the EC barrier permeability through the RAGE/ROCK1 pathway in the early stage.

## Methods

### Reagents

Human pulmonary microvascular endothelial cell (HPMEC) was obtained from Tongpai Biotechnology Co., Ltd (Shanghai, China). Recombinant human HMGB1 (rhHMGB1) was obtained from Shanghai Primegene Bio-Tech Co., Ltd (Shanghai, China). FPS-ZM1 (a high-affinity RAGE-specific inhibitor) was from Sigma-Aldrich (St. Louis, MO, USA). Y-27632 (ROCK inhibitor) was from Selleck Chemicals (Houston, Texas, USA). CCK-8 kit was from Beyotime Technology (Shanghai, China). Fluorescein isothiocyanate-dextran was from Sigma-Aldrich (St. Louis, MO, USA). Phalloidin-iFluor 594 Conjugate was obtained from Absin Bioscience (Shanghai, China). ROCK1/2 siRNA (SC-29473/SC-29474), RAGE siRNA (SC-36374) and negative control siRNA were from Santa Cruz Biotechnology Co., Ltd (Shanghai, China). Lipofectamine^®^ Reagent was employed for siRNA transfection (Paisley, UK). ZO-1 (monoclonal rabbit anti-human, CST 13663S), VE-cadherin (monoclonal rabbit anti-human, CST 2500T), MLC (monoclonal rabbit anti-human, CST 3672), p-MLC (Ser-19, monoclonal rabbit anti-human, CST 3674T) and ROCK1/2 (monoclonal rabbit anti-human, CST 4035/9029) antibodies were obtained from Cell Signaling Technology (CST, Boston, USA). RAGE (polyclonal rabbit anti-human, Cat No. 16346-1-AP) and Tubblin (monoclonal mouse anti-human, Cat No. 66240-1-Ig) antibodies were from Proteintech (North America). RhoA antibody (monoclonal rabbit anti-human, Catalog # 17-294 Lot # DAM1764537) was from Millipore Corporation (Temecula, CA). Rho activation kit was purchased from Upstate Biotechnology (Millipore, Dundee, UK). Anti-mouse and anti-rabbit secondary antibodies conjugated to horse radish peroxidase were purchased from Proteintech (North America). Donkey anti-Rabbit IgG (H+L) Alexa Fluor 488 Highly Cross-Adsorbed Secondary Antibody was from Invitrogen (Carlsbad, USA). Regular Range Prestained Protein Marker was from Proteintech (North America). Enhanced chemiluminescence (ECL) was from Tanon Science & Technology Co. (Shanghai, China).

### Cell Culture and Treatments

HPMECs were inoculated in Dulbecco’s modified Eagle’s culture medium with 10% fetal bovine serum at 37°C and in 5% CO_2_. The medium was replaced every 1-2 days. All experiments were conducted in confluent monolayers on the 3rd or 4th day after seeding (passages 5-7). After 24 hours of serum-free culture, the cells were treated with rhHMGB1. rhHMGB1 was trypsinized to abolish the micro-amounts of endotoxin (7). HPMECs were then stimulated with rhHMGB1 at 600 ng/ml for 10 min, 30 min, 1 h, 6 h and 24 h, or treated with rhHMGB1 at 100, 200 and 600 ng/ml for 24 h. In order to detect the toxicity of Y-27632 and FPS-ZM1 to ECs, HPMECs were treated with Y-27632 (5, 10, 15 uM) and FPS-ZM1 (0.01, 0.05, 0.1 uM) for 24 h respectively.

### Transfection of siRNA

HPMECs were grown on dishes precoated with 4 g/ml fibronectin. To prepare siRNA-lipofectamine complexes, siRNAs were mixed with lipofectamine reagent diluted in OptiMEM^®^ medium for 5 min at room temperature. ECs at 60-80% confluence were treated for 4 h with 10 nM ROCK1/2 siRNA, 100 nM RAGE siRNA or the corresponding negative control siRNA through adding the siRNA lipofectamine complexes to the ECs in serum-free medium. The transfected cells were then incubated with normal medium at 37°C with 5% CO2 for 48 h.

### Cell Viability Assay

Cell viability was examined by CCK-8 measurement. HPMECs were inoculated in 96-well plates. CCK-8 solution (10 μl/well) was added, followed by culture at 37°C for 4 h. The absorbance at 450 nm was detected with a microplate reader (Thermo Labsystems, IL, USA). Cell viability was calculated as a percentage of that of control group.

### Measurement of Endothelial Permeability

HPMECs were inoculated on 0.4-um pore Transwell filters. FITC-dextran (MR 40,000; Sigma–Aldrich) was added into the upper chamber at a concentration of 1 mg/ml and equalized for 1h, then the culture medium sample was collected from the lower chamber to detect base permeability. After indicated treatment, the media were taken from the lower chamber. The FITC fluorescence intensity was detected by a fluorescence spectrometer (MV06744, MoleCular Devices, Shanghai). The excitation wavelength was 482 nm and the detection wavelength was 525 nm.

### Immunofluorescence

For VE-cadherin and ZO-1 localization, ECs were fixed with ice-cold methanol on chamber slides. Cells were blockaded with serum and treated with primary antibodies (VE-cadherin and ZO-1, 1:200) and Alexa Fluor 488 donkey anti-rabbit antibody (1:200). For F-actin localization, ECs were fixed in 4% paraformaldehyde, permeabilized with 0.1% Triton X-100, blockaded with 5% BSA, and treated with 5 mg/ml of fluorescein isothiocyanate conjugated phalloidin. Confocal laser scanning microscope was used for image acquisition (Zeiss, Germany). The fluorescence intensity of F-actin, VE-cadherin and ZO-1 was quantitatively analyzed by the Image J software (National Institutes of Health, Bethesda, MD, USA).

### Western Blot

Protein concentration was measured by the BCA method, and then the samples were titrated to the same concentration. Protein samples (10 μl) were subjected to 10% SDS-PAGE, transferred to a polyvinylidene fluoride membrane, blockaded by 5% BSA at room temperature for 1 h, then treated with primary antibodies (4°C, overnight) followed by incubation with HRP-coupled anti-mouse/rabbit IgG antibody (1:8000 dilution, room temperature, 1 h). Bands were developed with SuperSignalWest Pico Chemiluminescent Substrate and images were captured by Tanon 5200 System (Tanon, Shanghai). Primary antibodies and their dilution ratios applied in this present study were as follows: anti-RhoA (1:2000), anti-ROCK1/2 (1:1000), anti-MLC (1:1000), anti-pMLC (Ser-19, 1:1000), anti-VE-cadherin (1:1000), anti-ZO-1 (1:1000), anti-RAGE (1:800) and anti-tubblin (1:8000). Anti-tubblin protein was determined as an endogenous control for other proteins. At least three different repeats were performed for quantification. Band intensity was normalized by its own endogenous control.

### Assessment of Activated RhoA

RhoA activation was determined with a pull-down assay kit in line with the manufacturer’s instructions. ECs were lysed with a Triton X-100 lysis buffer. EC lysates were centrifuged at 13,000g at 4°C for 3 min, and equal volumes of lysates were treated with rhotekin-Rho-binding domain-coated agarose beads at 4°C for 1 h, then the beads were washed three times. The content of GTP-RhoA (RhoA associated with the beads) and total RhoA in cell lysates were measured by immunoblot. The activity of RhoA was examined by normalizing the amount of rhotekin-Rho-binding domain-bound RhoA to the total amount of RhoA in cell lysates.

### Statistical Analysis

Experiments were performed in a minimum of triplicate replications. All values were expressed as means ± standard deviation (SD). SPSS 17 for Windows was used for statistical analysis. The one-way ANOVA test followed by Dunnett’s post-test was applied for comparisons between groups. *P* value < 0.05 was considered to indicate statistical significance.

## Results

### HMGB1-Mediated the Formation of Stress Filaments and Disruption of AJ/TJ Proteins

**Figure 1:**
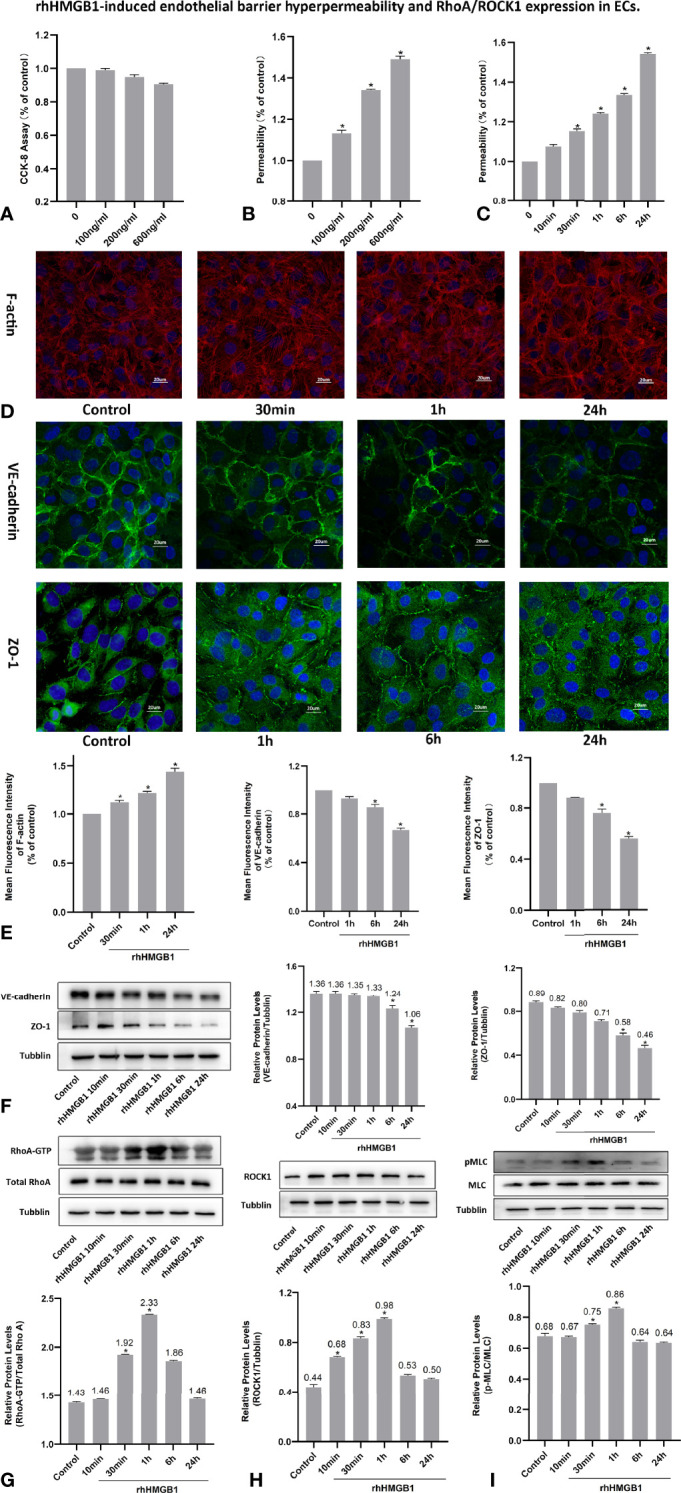
rhHMGB1-induced endothelial barrier hyperpermeability and RhoA/ROCK1 expression in ECs. **(A)** Cell viability of HPMEC was evaluated by CCK-8 measurement after stimulated with different concentration of rhHMGB1 for 24 h. **(B)** HPMECs were stimulated with the indicated concentrations of rhHMGB1 for 24 h. **(C)** HPMECs were stimulated with 600 ng/ml rhHMGB1 for the indicated times. **(D, E)** Immunofluorescence location of F-actin, VE-cadherin and ZO-1 in HPMECs was detected after 600 ng/ml rhHMGB1 stimulation for the indicated times. The fluorescence intensity of F-actin, VE-cadherin and ZO-1 was quantitatively analyzed using the Image J software. **(F)** The concentration of 600 ng/ml rhHMGB1 could selectively downregulate the expression level of VE-cadherin and ZO-1 at 24 h. **(G)** Time course of rhHMGB1-mediated increase in RhoA activity. Western blots showed the content of GTP-bound RhoA and total RhoA in cell lysate. **(H)** rhHMGB1 (600 ng/ml) treatment significantly upregulated ROCK1 expression in HPMECs at 60 min. **(I)** Treatment with 600 ng/ml rhHMGB1 could transiently promote the expression of pMLC. Values were shown as mean ± SD of 3 independent trials. *p < 0.05 *vs*. control.

**Figure 2:**
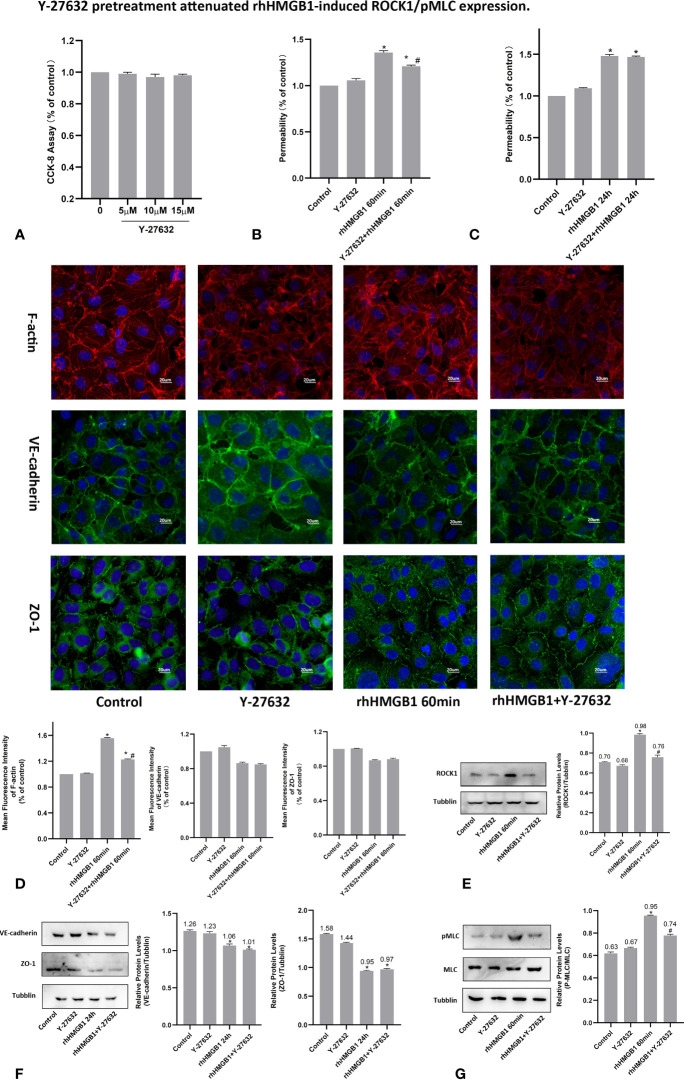
Y-27632 pretreatment attenuated rhHMGB1-induced ROCK1/pMLC expression. **(A)** CCK-8 assay was performed with HPMECs for 24 h with different dosages of Y-27632 as indicated. **(B)** Effects of Y-27632 on changes in FITC-dextran flux in HPMECs. HPMECs were pretreated with Y-27632 and then treated with 600 ng/ml rhHMGB1 for 60 min. **(C)** Role of Y-27632 pretreatment in increased barrier permeability induced by rhHMGB1 at 24 h. **(D)** Effects of Y-27632 on rhHMGB1-mediated morphological change in endothelial F-actin, VE-cadherin and ZO-1. HPMECs were pretreated with Y-27632 for 1 h before rhHMGB1 (600 ng/ml) stimulation for 60 minutes to examine morphology of endothelial F-actin, VE-cadherin and ZO-1 by immunofluorescence. Fluorescence intensity of F-actin, VE-cadherin and ZO-1 was measured in ECs. **(E)** Y-27632 pretreatment downregulated the ROCK1 expression induced by rhHMGB1 at 60 min. **(F)** Effects of Y-27632 treatment on rhHMGB1-induced changes in the protein expression levels of VE-cadherin and ZO-1 at 24 h. Y-27632 were added for the last 4 h of the 24 h rhHMGB1 treatment. **(G)** Pretreatment with Y-27632 attenuated rhHMGB1-induced MLC phosphorylation at 60 min. ECs were pretreated with Y-27632 for 1 h and then stimulated with rhHMGB1 (600 ng/ml) for 1 h. Values were indicated as mean ± SD of 3 separate trials. *p < 0.05 *vs*. control. ^#^ *p* < 0.05 *vs*. rhHMGB1 60-min group.

The changes of cell viability in HPMECs treated with different concentrations of rhHMGB1, Y-27632 and FPS-ZM1 were examined by CCK-8 method (**Figures 1A**, **2A** and **4A**). As indicated in **Figures 1B, C**, rhHMGB1 stimulation upregulated FITC-dextran flux in a dose-dependent and time-dependent manner. rhHMGB1 at a dose of 600 ng/ml showed a significant effect on EC barrier permeability (**Figure 1B**). The barrier permeability of HPMEC was markedly increased 30 min after rhHMGB1 treatment, and progressively increased to 24 h (**Figure 1C**). Therefore, the selected concentration of rhHMGB1 was 600 ng/ml in the following experiments. ECs were also treated with Y-27632 (10 uM) and FPS-ZM1 (0.05 uM) for 1 h prior to stimulation with rhHMGB1 and for the last 4 h of the 24 h rhHMGB1 stimulation respectively. Immunofluorescence microscopy revealed that rhHMGB1 also induced progressive cytoskeletal changes in cultured HPMECs that were apparent after 30 min of rhHMGB1 treatment (**Figure 1D**). The 24 h exposure of rhHMGB1 elicited the formation of stress filaments and paracellular gaps (**Figures 1D, E**). After rhHMGB1 treatment for 60 min, membrane localization of VE-cadherin and ZO-1 was also significantly ruptured, indicating that AJ/TJ integrity was disrupted (**Figure 1E**). To investigate the molecular mechanisms for the rhHMGB1-mediated endothelial barrier disruption, we checked whether changes in endothelial cell barrier permeability were paralleled with changes in the expression levels of intercellular junction proteins. As shown in **Figure 1F**, western blot revealed that treatment with rhHMGB1 elicited a time-dependent decrease in the expressions of VE-cadherin and ZO-1, which were measurable at 6 h of rhHMGB1 stimulation in ECs, and a more significant decrease was measured at 24 h.

### Effects of ROCK1 on HMGB1-Mediated HPMEC Hyperpermeability

**Figure 3:**
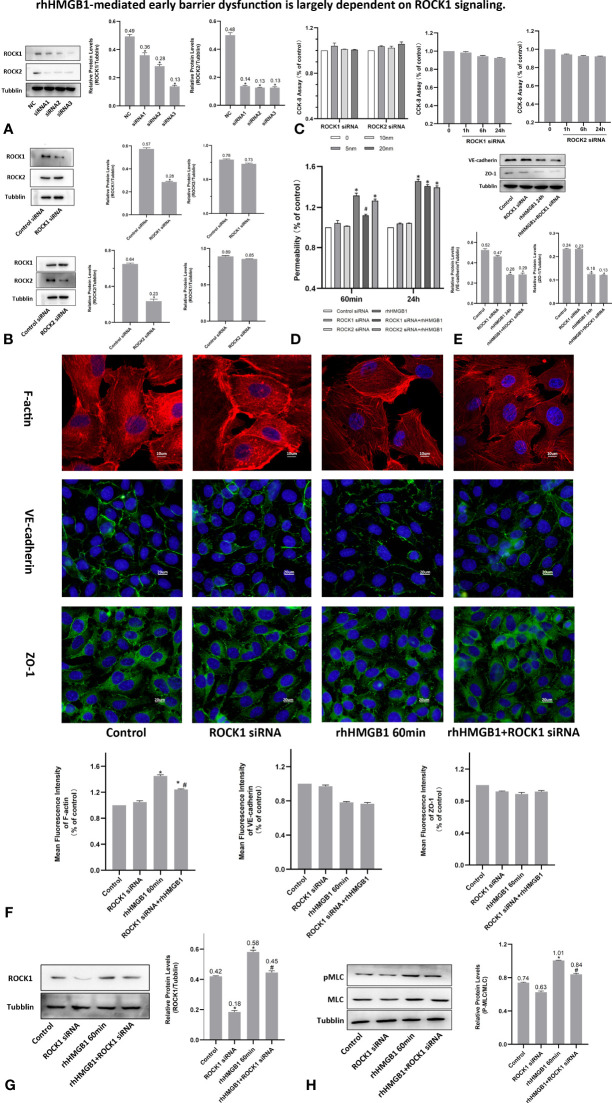
rhHMGB1-mediated early barrier dysfunction is largely dependent on ROCK1 signaling. **(A, B)** ECs were transfected with ROCK1/2 siRNA. Western blot was used to assess the protein expression of ROCK1/2 in HPMECs. **(C)** Cell viability was assessed by the CCK-8 assay after transfection with different concentration of ROCK1/2 siRNA for 24 h or transfection with 10 nM ROCK1/2 siRNA for the indicated times. There was no evidence of cytotoxicity found in ROCK1/2 siRNA transfected cells. **(D)** Examination of FITC-dextran flux of HPMECs. ROCK1 knockdown ameliorated rhHMGB1-induced early permeability increases (at 60 min). **(E)** Effects of ROCK1 siRNA on the expression of VE-cadherin and ZO-1 induced by rhHMGB1 at 24 h. **(F)** ECs were transfected with ROCK1 siRNA and then stimulated with rhHMGB1 for 60 min. Immunofluorescence staining of F-actin, VE-cadherin and ZO-1 was detected by fluorescence microscopy. Image J software was used to analyze the fluorescence intensity of F-actin, VE-cadherin and ZO-1. **(G)** ROCK1 knockdown attenuated the ROCK1 expression induced by rhHMGB1 at 60 min. **(H)** ROCK1 knockdown downregulated the rhHMGB1-induced pMLC expression in cells at 60 min. Mean ± SD of 3 independent trials was shown. *p < 0.05 *vs*. corresponding control group. ^#^ *p* < 0.05 *vs*. rhHMGB1 60-min group. NC, negative control.

To investigate the effects of ROCK activation played on the rhHMGB1-mediated EC barrier hyperpermeability, the ECs were pretreated with Y-27632 or transfected with ROCK1/2 siRNA before rhHMGB1 stimulation. Western blot was used to assess the protein expression of ROCK1/2 in HPMECs after transfection with ROCK1/2 siRNA and there was no evidence of cytotoxicity found in ROCK1/2 siRNA transfected cells (**Figures 3B, C**). As shown in **Figures 1G, H**, the time-dependent increases in RhoA activity and ROCK1 expression by rhHMGB1 treatment were measured. The activity of RhoA/ROCK1 was significantly upregulated at 30 min and 60 min of rhHMGB1 treatment, but the activity of RhoA/ROCK1 returned to baseline by 24 h. The rhHMGB1-induced EC hyperpermeability at 60min was significantly inhibited by Y-27632 pretreatment and ROCK1 knockdown (**Figures 2B**, **3D**). However, transfection with ROCK2 siRNA alone did not reduce rhHMGB1-induced early permeability increases at 60 min (**Figure 3D**). In addition, transfection with ROCK1/2 siRNA had no significant inhibitory role in rhHMGB1-induced late permeability increases at 24 h (**Figure 3D**). When ECs were treated with Y-27632 for the last 4 h of the 24 h rhHMGB1 stimulation, the results indicated that suppression of ROCK had no marked inhibitory role in rhHMGB1-induced hyperpermeability at 24 h (**Figure 2C**), in accordance with the presence of stress filaments and intercellular gaps at 24h (**Figures 1D, E**). These data indicated that ROCK1 activation was necessary for rhHMGB1-mediated early increase in endothelial barrier permeability. Furthermore, the results of western blot showed that treatment with Y-27632 and ROCK1 siRNA had no significant effects on the expressions of VE-cadherin and ZO-1 in HPMECs after 24 h of rhHMGB1 stimulation (**Figures 2F**, **3E**).

### HMGB1 Induced MLC Activation in HPMECs

**Figure 4:**
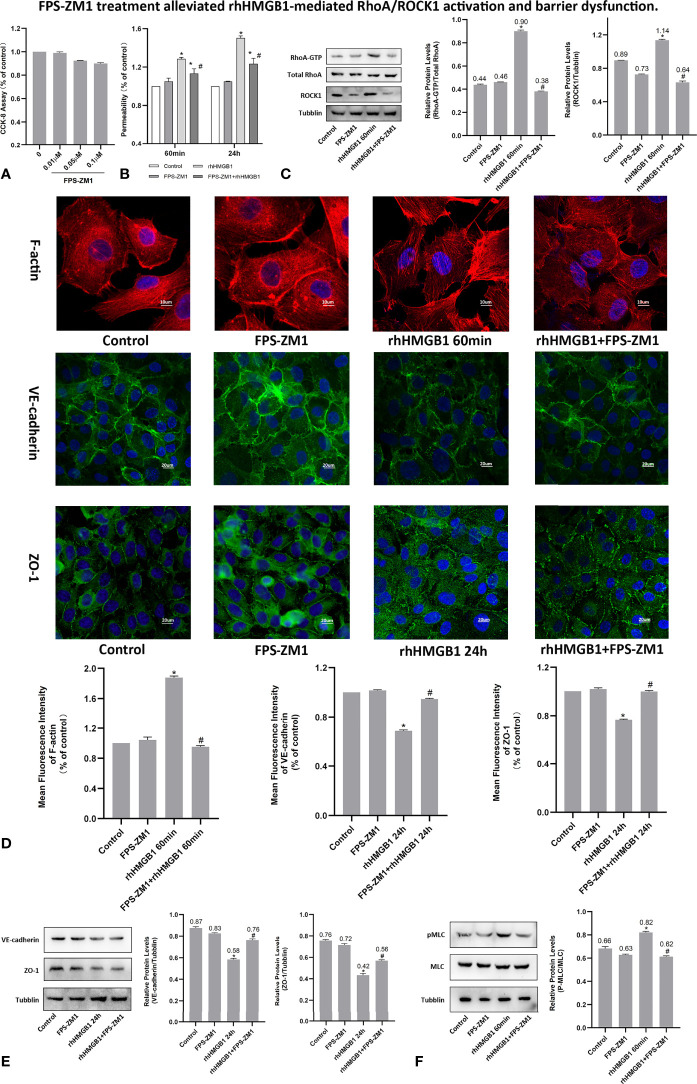
FPS-ZM1 treatment alleviated rhHMGB1-mediated RhoA/ROCK1 activation and barrier dysfunction. **(A)** Cell viability was determined by CCK-8 assay after treated with different dosage of FPS-ZM1 for 24 h. **(B)** FPS-ZM1 improved lung endothelial permeability at 60 min and 24 h after rhHMGB1 stimulation. **(C)** FPS-ZM1 significantly downregulated RhoA and ROCK1 expression in HPMECs at 60 min after rhHMGB1 stimulation. **(D)** Effects of FPS-ZM1 on rhHMGB1-mediated morphological changes in endothelial F-actin, VE-cadherin and ZO-1. ECs were treated with FPS-ZM1 for 1 h prior to stimulation with rhHMGB1 to evaluate morphology of endothelial cytoskeleton F-actin or for the last 4 h of the 24 h rhHMGB1 stimulation to assess morphology of endothelial VE-cadherin and ZO-1 by immunofluorescence microscopy. Image J software was used to analyze the fluorescence intensity of F-actin, VE-cadherin and ZO-1. **(E)** FPS-ZM1 significantly increased VE-cadherin and ZO-1 expression levels in HPMECs at 24 h after rhHMGB1 stimulation. ECs were treated with FPS-ZM1 for the last 4 h of the 24 h rhHMGB1 stimulation. **(F)** Effects of FPS-ZM1 on rhHMGB1-mediated pMLC expression in cells. ECs were pretreated with FPS-ZM1 for 1 h and then treated with rhHMGB1 for 60 minutes. Mean ± SD of 3 independent trials was shown. *p < 0.05 *vs*. control. ^#^ *p* < 0.05 *vs*. rhHMGB1 60-min group or rhHMGB1 24-h group.

**Figure 5:**
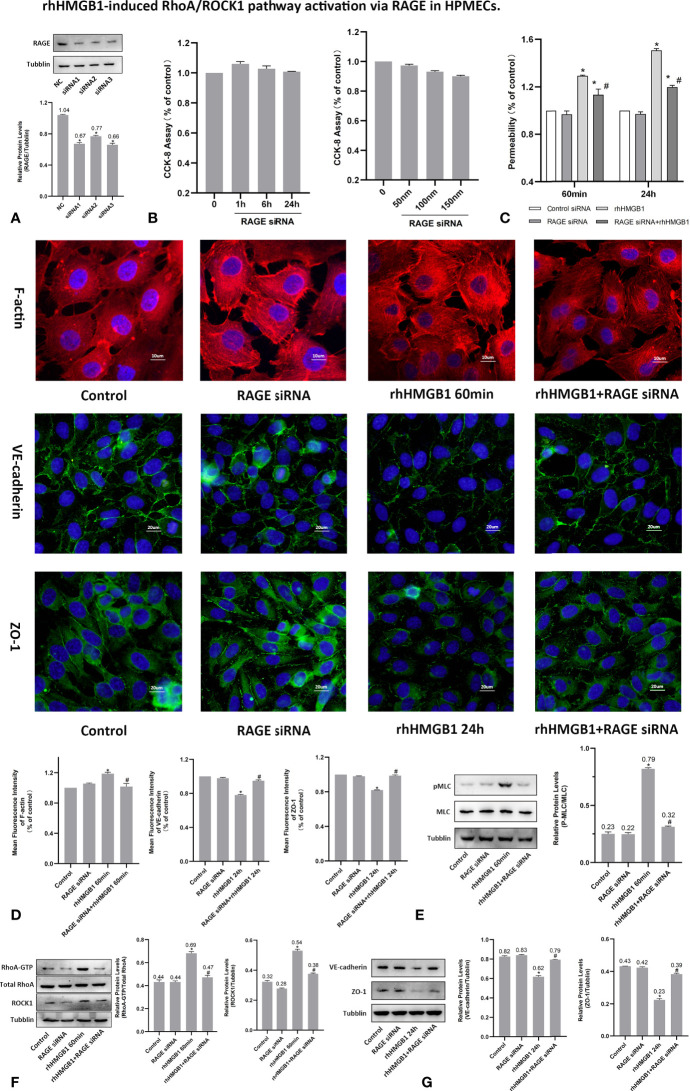
rhHMGB1-induced RhoA/ROCK1 pathway activation *via* RAGE in HPMECs. **(A)** ECs were transfected with RAGE siRNA. Western blots were used to determine the expression of RAGE in endothelial cells. **(B)** Cytotoxicity of RAGE siRNA was assessed by CCK-8 assay after transfected with different concentration of RAGE siRNA for 24 h or transfected with 100 nM RAGE siRNA for the different times. No evidence of cytotoxicity was found in RAGE siRNA transfected cells. **(C)** Treatment with RAGE siRNA ameliorated endothelial barrier dysfunction induced by rhHMGB1 at 60 min and 24 h. **(D)** ECs were transfected with RAGE siRNA and then stimulated with rhHMGB1 for 60 min and 24 h. Immunofluorescence staining of F-actin, VE-cadherin and ZO-1 was determined by fluorescence microscopy. Fluorescence intensity of F-actin, VE-cadherin and ZO-1 was measured in ECs. **(E)** Knockdown of RAGE by siRNA reduced the rhHMGB1-induced MLC phosphorylation at 60 min as detected by western blot. **(F)** Effects of inhibition of RAGE with siRNA on increased expression of RhoA and ROCK1 induced by rhHMGB1 at 60 min in HPMECs. **(G)** Role of RAGE siRNA in the VE-cadherin and ZO-1 protein expression levels in ECs at 24 h after rhHMGB1 treatment. Mean ± SD of 3 independent trials was shown. *p < 0.05 *vs*. corresponding control group. ^#^ *p* < 0.05 *vs*. rhHMGB1 60-min group or rhHMGB1 24-h group. NC, negative control.

It was demonstrated that Rho/ROCK signaling pathway played a key influence on increasing the level of MLC phosphorylation (19). Thus, pMLC is an important initial event for the increased paracellular flow of endothelial cell leakage (18). In the present study, some modest morphological changes occurred in the actin cytoskeleton after treatment with rhHMGB1 for 30 min (**Figure 1D**), which could reflect the enhanced Rho/ROCK activity and MLC phosphorylation. As shown in **Figure 1I**, rhHMGB1 stimulation (600 ng/ml) for 60 minutes obviously increased the expression level of pMLC, which was consistent with a time-dependent increase in RhoA activity and ROCK1 expression (**Figures 1G, H**). Results showed that rhHMGB1 induced an increase in Ser-19 phosphorylation at 30 min and 1 h and the expression level of Ser-19 phosphorylated MLC returned to baseline by 24 h, as shown on immunoblot (**Figure 1I**). The inhibition of ROCK1 expression with Y-27632 and ROCK1 siRNA could downregulate the phosphorylation of MLC after 60 min of rhHMGB1 treatment (**Figures 2G**, **3H**), which was accompanied by decreases in the fluorescence intensity of F-actin at 60 min (**Figures 2D**, **3F**). Pretreatment with FPS-ZM1 and RAGE siRNA could result in a marked downregulation of the activity of RhoA/ROCK1 and phosphorylation of MLC in HPMECs treated with rhHMGB1 for 60 minutes (**Figures 4C, F** and **5E, F**), and then reduce the fluorescence intensity of stress fibers in the cell center at 60 min (**Figures 4D**, **5D**).

### RhoA/ROCK1 Pathway Mediates HMGB1-Induced EC Barrier Disruption in HPMECs

As shown in **Figures 1G, H**, the time-dependent increases in RhoA activity and ROCK1 expression by rhHMGB1 treatment were measured. The results showed that the peak activity of RhoA appeared at 60 min and rhHMGB1 treatment also significantly up-regulated the expression of ROCK1 at 60min. Thus, binding of active GTP-bound RhoA caused unfolding and activation of ROCK1, which was accompanied by rhHMGB1-mediated MLC phosphorylation, stress filament formation, VE-cadherin and ZO-1 disruption, and the early increase in EC barrier permeability (**Figure 1**). Pretreatment with Y-27632 and ROCK1 knockdown could partially inhibit rhHMGB1-mediated EC leakage and restore FITC-dextran flux of the endothelial barrier after 60 min of rhHMGB1 stimulation (**Figures 2B**, **3D**). After pretreatment with Y-27632 and ROCK1 siRNA, the expression of ROCK1 protein at 60 min was significantly downregulated (**Figures 2E**, **3G**), and the membrane location of VE-cadherin and ZO-1 in intercellular junctions was partially restored after 60 min of rhHMGB1 stimulation (**Figures 2D**, **3F**). The 60 min exposure of rhHMGB1 increased the fluorescence intensity of F-actin in the cell center, and pretreatment with Y-27632 and ROCK1 siRNA decreased the fluorescence intensity of central stress filaments after 60 min of rhHMGB1 exposure (**Figures 2D**, **3F**). These findings suggest that RhoA and ROCK1 may be implicated in rhHMGB1-mediated early increase in EC permeability and stress filament formation in HPMECs.

### RAGE Mediates the EC Barrier Leakage Induced by HMGB1

To investigate whether RAGE is implicated in the rhHMGB1-mediated increases in EC barrier permeability, the expression of RAGE was silenced by siRNA in HPMECs. In addition, HPMECs were also treated with the specific RAGE inhibitor FPS-ZM1 (20) for 60 min prior to stimulation with rhHMGB1 or for the last 4 h of the 24 h rhHMGB1 stimulation. As indicated in **Figures 4B**, **5C**, FPS-ZM1 and RAGE knockdown significantly decreased endothelial permeability by comparison with the rhHMGB1 treatment group at 60 min and 24 h. Inhibition of RAGE with FPS-ZM1 and RAGE siRNA could also downregulate the activity of RhoA/ROCK1 and phosphorylation of MLC at 60 min (**Figures 4C, F** and **5E, F**), and then partly prevent the rhHMGB1-mediated formation of stress fibers in the cell center (**Figures 4D**, **5D**). Furthermore, inhibition of ROCK1 could also reduce the expression of pMLC (**Figures 2G**, **3H**), and partly recover the membrane localization of VE-cadherin and ZO-1 after 60 min of rhHMGB1 stimulation (**Figures 2D**, **3F**). Therefore, it seems likely that a signaling pathway proceeds from RAGE to RhoA/ROCK1 through the F-actin reorganization and disruption of AJ/TJ related proteins, which finally destroys the early EC barrier function in the present study. In addition, FPS-ZM1 and RAGE siRNA could upregulate the expression levels of VE-cadherin and ZO-1 at 24 h (**Figures 4E**, **5G**). Similarly, inhibition of RAGE could also partially restore the cytomembrane location of VE-cadherin and ZO-1 after 24 h of rhHMGB1 stimulation (**Figures 4D**, **5D**).

## Discussion

HMGB1 is released in late endotoxemia and it is closely associated with the severity and prognosis of sepsis (21, 22). It is demonstrated that HMGB1 could elicit microvascular EC cytoskeletal rearrangement and barrier dysfunction (12). EC cytoskeleton, especially F-actin rearrangement, is main histological basis in enhanced EC barrier permeability, which elicits the increase in cell contractility and intercellular gap formation (23, 24). To our best knowledge, this study demonstrates here that HMGB1 elicits progressive changes to the filamentous actin, intercellular junctions and increases HPMEC barrier permeability in a time-dependent and dose-dependent manner. Furthermore, RAGE and RhoA/ROCK1 were implicated in the HMGB1-mediated EC cytoskeletal reorganization and early endothelial barrier dysfunction. This present study showed that HMGB1 disrupted the microvascular endothelial cell barrier, which might be implicated in the pathogenesis of ALI in sepsis.

Phosphorylation of MLC has been reported to involve in the regulation of EC barrier permeability after treatment with thrombin, histamine, and other inflammatory cytokines and so on (18, 19). In the present study, the early rhHMGB1-induced MLC phosphorylation and subsequent formation of actin stress fibers indicated that changes in EC contractility was occurring. RhoA plays a key role in control of endothelial cellular actin cytoskeletal rearrangement and cell morphology. ROCK (a downstream target of RhoA) mediates stress filament formation by upregulating the levels of MLC phosphorylation (25–27). Then, phosphorylated MLC contributes to actomyosin interaction, causing EC contraction and an increased permeability (28, 29). Furthermore, ROCK1 activation has been reported to influence cell-cell adhesion by modulating interactions between stress fibers and intercellular binding molecules (TJ and AJ) (30). It was demonstrated that ROCK1 activation induced by high glucose caused endothelial-to-mesenchymal transition with loss of CD31 and VE-cadherin, resulting in increased endothelial permeability (31). Previous study also shows that anthrax lethal toxin causes EC barrier dysfunction through actin filament formation and disruption of adherens junctions (32). This study indicated that the changes of VE-cadherin expression associated temporally with the formation of stress fibers and activation of ROCK1 (32).

In this study, our findings showed that RhoA/ROCK1 was activated rapidly by rhHMGB1 in cultured HPMECs and rhHMGB1 stimulation induced rapid aggregation of actin stress filaments, intercellular gap formation and a significant increase in endothelial cell permeability by 60 minutes, indicating that the early cytoskeletal reorganization could affect permeability. To confirm the role of ROCK played in rhHMGB1-mediated EC barrier dysfunction, Y-27632 was added for 1 h prior to treatment with rhHMGB1 or for the last 4 h of the 24 h rhHMGB1 stimulation. Moreover, the ECs were transfected with ROCK1/2 siRNA before rhHMGB1 treatment. The results showed that Y-27632 and ROCK1 knockdown were able to significantly suppress ROCK1 activation, subsequent MLC Phosphorylation and the rhHMGB1-induced hyperpermeability at 60 min. In addition, Y-27632 and ROCK1 knockdown decreased the number of central stress filaments and partially restored cortical localization of F-actin and membrane location of VE-cadherin and ZO-1 at 60 min of rhHMGB1 stimulation in HPMECs. Increased endothelial barrier permeability is often related to lack or disruption of AJ/TJ proteins (7, 33) and indeed VE-cadherin and ZO-1 expressions were downregulated following a long-term rhHMGB1 stimulation in this study. The results of western blot showed that inhibition of ROCK with Y-27632 had no influence on the expression of VE-cadherin and ZO-1 at 24 h of rhHMGB1 stimulation in HPMECs, suggesting that rhHMGB1 induced the disruption of EC barrier at 24 h independent of its effect on the RhoA/ROCK signaling. In addition, our previous study indicated that HMGB1 downregulated AJ/TJ components at 24 h through activation of the RAGE/p38 signaling pathway (7). Other study showed that HMGB1 elicited the activation of the RAGE/ERK1/2 pathway at 24 h, which correlated with barrier dysfunction in the human bronchial epithelial cells (34).

So far, many evidences show that HMGB1 interacts with endothelial cell through RAGE. To study the role of RAGE in EC barrier dysfunction, we inhibited the RAGE activity using RAGE siRNA and its specific inhibitor FPS-ZM1 (20, 35). Recent studies indicated that the inhibition of RAGE functions *via* FPS-ZM1 might be a meaningful therapeutic strategy for a variety of diseases, such as diabetes-related glomerular filtration barrier damage and irradiation-induced EC barrier disruption (10, 36). Our study also demonstrated that the blockage of RAGE with FPS-ZM1 and RAGE siRNA attenuated the EC barrier hyperpermeability mediated by rhHMGB1 and blocked the rhHMGB1-induced RhoA/ROCK1 activation and MLC phosphorylation. Furthermore, our findings also confirmed FPS-ZM1 as a potential therapeutic drug for treating rhHMGB1-induced EC barrier dysfunction.

In conclusion, our findings demonstrate that rhHMGB1 could induce the early EC barrier disruption, and the potential molecular mechanism may be that rhHMGB1 activates the RhoA/ROCK1 signaling pathway through RAGE, which mediates the phosphorylation of MLC inducing stress fiber formation at short time (up to 60 min), and HMGB1/RAGE disrupts the integrity of AJ/TJ at long term (up to 24 h) independently of RhoA/ROCK1 signaling pathway. These new findings will help to understand the signaling pathways of rhHMGB1-mediated increase in EC barrier permeability and contribute to establish potential therapeutic targets in the treatment of sepsis.

## Data Availability Statement

The original contributions presented in the study are included in the article/supplementary material. Further inquiries can be directed to the corresponding authors.

## Author Contributions

M-JZ, H-RJ, J-WS, Z-AW, BH, C-RZ, X-HY, M-MC and X-CM participated in experimental design, research, data analysis and draft writing. M-JZ, H-RJ and Z-GL wrote and revised the manuscript. Z-GL and W-DZ contributed to the research concept, study design, data analysis and finalization. All authors contributed to the article and approved the submitted version.

## Funding

The present study was supported by grants from the Natural Science Foundation of Liaoning Province, China (No. 2019-MS-09) and the Liaoning Xingliao Talent Plan Project (No. XLYC2005015).

## Conflict of Interest

The authors declare that the research was conducted in the absence of any commercial or financial relationships that could be construed as a potential conflict of interest.

## Publisher’s Note

All claims expressed in this article are solely those of the authors and do not necessarily represent those of their affiliated organizations, or those of the publisher, the editors and the reviewers. Any product that may be evaluated in this article, or claim that may be made by its manufacturer, is not guaranteed or endorsed by the publisher.

## Abbreviations

HPMEC, human pulmonary microvascular endothelial cell; HMGB1, high mobility group box 1; ALI, acute lung injury; AJ, adherens junction; TJ, tight junction; VE-cadherin, vascular endothelial cadherin; ZO-1, zonula occludens 1; RAGE, receptor for advanced glycation end products; ROCK, Rho kinase; MLC, myosin light chain; pMLC, phosphorylated MLC; EC, endothelial cell.
